# Two approaches for repeat cardiac surgery

**DOI:** 10.1186/1749-8090-7-114

**Published:** 2012-10-22

**Authors:** Jin Woo Chung, Hyun Keun Chee, Jun Seok Kim

**Affiliations:** 1 Department of Thoracic and Cardiovascular Surgery, Konkuk University Medical Center, Konkuk University School of Medicine, 4-12, Hwayang-dong, Gwangjin-gu, Seoul, 143-701, South Korea

**Keywords:** Reoperation, Sternotomy, Thoracotomy

## Abstract

**Background:**

With recent advances in post-operative care and surgical methods, the number of cardiovascular re-operations is increasing. We analyzed our institutional experience to evaluate the safety and efficacy of the approach methods for cardiac re-operations.

**Methods:**

Between September 2007 and December 2010, we performed 208 cardiac re-operations, defined as surgery which was not performed within a month from the previous operation or during the same hospitalization for the same disease and reviewed retrospectively. According to the surgical approach, we divided patients into two groups: median sternotomy group (S-group, n = 146), and thoracotomy group (T-group, n = 62).

**Results:**

There were no differences in sex or mean interval from the first surgery to re-operation between the two groups. Mean cardiopulmonary bypass, adhesion dissection time, bleeding control time, and operation time were significantly shorter in the T-group. The need for transfusion (p = 0.001) during adhesion dissection and the chest tube drainage (p < 0.001) were significantly lower in the T-group. There were 10 operative deaths in the S-group (6.8%) and 5 in the T-group (8.1%) (p = 0.757). Pneumonia was the most common cause of death in both groups. Post-operative bleeding did not result in death and there were no cases of wound infection in the T-group.

**Conclusions:**

Two approaches for repeated cardiac surgery were safe and effective in terms of mortality, wound infection, bleeding, operation time, adhesion dissecting time, and bleeding control time. We were able to obtain a good visual field and perform safe surgery by applying the thoracotomy method in selective patients for cardiovascular re-operation.

## Background

With recent advances in post-operative care and surgical methods, the number of cardiovascular re-operations is increasing. The traditional median sternotomy is commonly considered the standard and popular approach for cardiovascular re-operation. However, when re-operations are performed through median sternotomy, problems unrelated to the underlying heart disease can occur, such as damage to the cardiac structure, prolonged duration of cardiopulmonary bypass, and increased need for blood transfusion [1-3]. The thoracotomy approach has been applied to avoid these risks, and recent studies showed that the thoracotomy approach for repeat cardiac surgery may decrease risks associated with cardiac surgery and improve early outcomes [4,5].

To address these issues, we analyzed our institutional experience to evaluate the safety and efficacy of two approach methods for cardiac re-operations.

## Methods

### Patients

Between September 2007 and December 2010, we performed 208 cardiac re-operations, defined as surgery which was not performed within a month from the previous operation or during the same hospitalization for the same disease and reviewed retrospectively. Mean age at re-operation was 59.0 ± 17.0 years; 91 patients were male and 117 were female. Mean interval from the first surgery to re-operation was 144.0 ± 113.0 months (median 119.7 months). One hundred seventy-one patients underwent a second operation, 28 patients had a third operation, and 9 patients had 4 or more operations. According to the surgical approach, we divided patients into two groups: median sternotomy group (S-group, n = 146), and thoracotomy group (T-group, n = 62). Only right thoracotomy was performed in the T-group. This study was approved by our institutional review board (No.: 1080015), and the requirement of informed consent was waived by the board owing to the retrospective nature of the study.

Table 1 reports reasons for re-operation. In the S-group, there were 68 cases of valvular heart disease, 33 congenital heart disease (pulmonary regurgitation after repair of tetralogy of Fallot in 25, residual shunt through ventricular septal defect in 2, others in 6), 21 aortic disease, 15 coronary artery disease, 3 dilated cardiomyopathy, and 6 other diseases. In the T-group, there were 59 cases of valvular heart disease, 2 congenital heart disease (residual shunt through atrial septal defect), and 1 other disease (Table 1).

**Table 1:** Causes of reoperation

	**Sternotomy group (146)**	**Thoracotomy group (62)**	**Total**
Aortic valve	33	0	33
Mitral valve	12	27	39
Tricuspid valve	1	19	20
Combined valve	15	8	23
Paravalvular leakage of prosthetic MV	7	5	12
Congenital	33	2	35
Aorta	21	0	21
Coronary	15	0	15
DCMP	3	0	3
Others	6	1	7

Abbreviation: MV, mitral valve; DCMP, dilated cardiomyopathy.

All patients except emergency cases underwent chest computed tomography to evaluate adhesion.

### Definition

Operation time was defined as the time from skin incision to skin closure. From the time of skin incision to the time of heparin injection was considered as the time of adhesion dissection. The interval between discontinuation of cardiopulmonary bypass and skin closure was regarded as the time of bleeding control.

### Surgical method

Our approach in cardiac re-operations is as follows. Right thoracotomy was performed on patients who needed re-operation due to atrioventricular valve diseases and/or residual shunt through atrial septal defect. Median sternotomy was performed for the patients with valvular heart disease including the aortic valve and/or aortic disease. We also performed the cardiac re-operation through median sternotomy when we could not gain an adequate approach via thoracotomy, such as pulmonary valve replacement after repair of tetralogy of Fallot, and heart transplantation.

In case of thoracotomy, we most commonly used the femoral artery, vein, and the right internal jugular vein as vascular access for cardiopulmonary bypass. The cannula through the right internal jugular vein was inserted percutaneously before beginning the operation. We used DLP cannulas (17 – 21 Fr. for artery, 21–24 Fr. for vein, Medtronic Inc., MN, USA) for femoral cannulation and RMI 20Fr. cannula (Edward’s lifescience LLC, Irvine, CA) for internal jugular cannulation. Thoracotomy was performed through the fourth intercostal space; the femoral artery and vein were prepared simultaneously. For female patients, skin incision was made along the crease line underneath the breast. A root cannula for infusion of cardioplegia and air ventilation was inserted in the ascending aorta. If a patient had patent grafts on the ascending aorta, we performed the surgery without aortic cross-clamp under fibrillatory arrest.

Median sternotomy was performed in the usual manner. We commonly used the ascending aorta, superior vena cava and inferior vena cava as vascular access for cardiopulmonary bypass. In case of severe adhesion between the mediastinum and the heart, we used the femoral artery and vein and superior vena cava as vascular access after we dissected adhesion as far as possible. If a patient had patent grafts on the ascending aorta, we performed aortic cross-clamp higher than level of the graft.

### Statistics

Data are presented as frequencies, or means with standard deviations. For the comparisons of patient characteristics between groups, student T-test was performed for continuous variable and chi-square test for categorical variables. A p value of 0.05 or less was considered significant. SPSS version 17 (Korean version) was used for statistical analysis.

## Results

Patient profiles and operation profiles were summarized in Table 2. Mean age at re-operation was younger in the S-group than the T-group (p = 0.004). There were no differences in sex or mean interval from the first surgery to re-operation between groups. Excluding 8 patients had undergone re-operation without cardiopulmonary bypass, the remaining 200 patients had a mean of 182 ± 94 minutes for the cardiopulmonary bypass. In the entire cohort, mean aortic cross clamping time was 113 ± 56 minutes, mean adhesion dissection time was 165 ± 63 minutes, mean bleeding control time was 130 ± 73 minutes, and mean operation time was 470 ± 160 minutes. T-group had shorter cardiopulmonary bypass time (p < 0.001), adhesion dissection time (p < 0.001), bleeding control time (p = 0.009) and operation time (p < 0.001). However, there was no difference in aortic cross clamping time (p = 0.069). In the T-group the need for red blood cell transfusion (1 pack = 400 ml) during adhesion dissection was significantly lower than in the S-group (p = 0.001). Chest tube drainage in the first 8 hrs was statistically lower in the T-group (p < 0.001).

**Table 2:** **Patient profiles and operationprofiles**

	**Sternotomy group**	**Thoracotomy group**	**P value**
Age, years	45.9 ± 18.0	53.4 ± 14.8	0.004
Sex(M/F), n	75/71	16/46	0.001
Operation interval, months	136.2 ± 117.0	162.2 ± 101.4	0.13
Operation time, minutes	502 ± 169	395 ± 106	<0.001
ACC time, minutes	119 ± 60	100 ± 37	0.069
CPB time, minutes	199 ± 99	144 ± 68	<0.001
Adhesion dissection time, minutes	177 ± 67	138 ± 44	<0.001
Bleeding control time, minutes	139 ± 83	110 ± 35	0.009
Mortality rate,%(n)	6.8% (10/146)^∮^	8.1% (5/62)^∮^	0.757
Hct_ind_,%	35.8 ± 4.6	35.8 ± 4.4	0.919
Hct_preCPB,_,%	33.2 ± 5.0	34.4 ± 4.7	0.103
RBC trasfusion, packs (1 pack = 400 ml)	0.5 ± 1.2	0.1 ± 0.5	0.001
Chest tube drainage(8 hrs), ml	466.2 ± 413.4	312.1 ± 199.2	<0.001

Abbreviation: M, male; F, female; ACC, aortic cross clamp; CPB, cardiopulmonary bypass; Hct_ind_, hematocrit just after anesthesia induction; . Hct_preCPB_, hematocrit just before the cardiopulmonary bypass institution; RBC, red blood cell.

**Figure 1:**
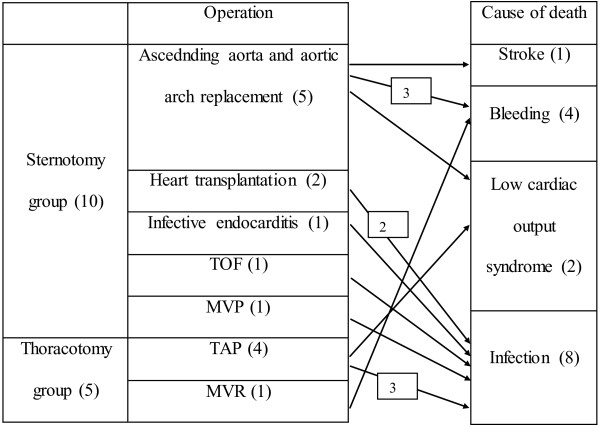
Causes of mortality.

There were 10 operative deaths in the S-group and 5 in the T-group. Causes of deaths were summarized in Figure 1. In the S-group, infections were most common cause of death. In the T-group, infections were also most common cause of death. Details of complications were summarized in Table 3. There were 23 complications overall. There were no differences between groups (p = 0.783). In the S-group, post-operative bleeding was most common complication. In the T-group, post-opeartive bleeding was also most common complication.

**Table 3:** Details of complications

	**Sternotomy group**	**Thoracotomy group**	**P value**
Total, n (%)	17 (11.6)	6(9.7)	0.783
Bleeding control, n	6	3	
Low cardiac output syndrome, n	5	1	
Infections, n	4	0	
Cerebrovascular accident, n	2	2	

In the T-group, all patients underwent peripheral cannulation using the femoral artery, vein and internal jugular vein for cardiopulmonary bypass. In the S-group, 46 patients underwent peripheral cannulation using the femoral artery and vein and superior vena cava and the others underwent central cannulation using the ascending aorta, superior vena cava and inferior vena cava for cardiopulmonary bypass. In the T-group, we did not perform aortic cross-clamp in 2 patients who underwent mitral valve surgery because of previously grafted vessels.32 patients underwent re-operation due to recurred tricuspid regurgitation. Ten patients were operated on via median sternotomy; the remaining 22 patients were approached by a right thoracotomy. There were no operative deaths in the S-group but four (12.5%) in the T-group.

## Discussion

The present study shows that both approach methods for cardiac re-operation show no significant differences in rates of operative mortality, post-operative bleeding, or cerebrovascular accidents. There was no cases of wound infection in the T-group. In this study, we included a variety of cardiac diseases and divided patients into two groups based on surgical approach. In order to evaluate the safety and efficacy of the approach methods, we only evaluated early results such as mortality and morbidity, because long-term follow-up results are more related to the underlying cardiac disease. For this reason we did not include long-term follow-up data.

Many re-operations are performed at sites of previous surgery. Deciding on the area of incision is of importance considering not only the surgery itself but also early outcome. The median sternotomy approach is commonly used for re-operation due to the advantage of a superior visual field. However, it carries an increased risk of ventricular rupture, brachial plexus damage from fracture of the first rib, and innominate vein injury. In the S-group, we instituted cardiopulmonary bypass after adhesion dissection to reduce the cardiopulmonary bypass time, but we performed peripheral cannulation before finishing adhesion dissection to avoid disastrous outcomes in 46 cases that had severe adhesion between the mediastinum and the heart. A unilateral thoracotomy approach minimizes peeling and dissection of adhesions in the surgical area, avoiding some of the risks associated with median sternotomy. There were 9 post-operative bleeding (4.3%) in the entire study population, with no significant difference between two groups. Operation time, cardiopulmonary bypass time, adhesion dissection time and bleeding control time were shorter in the T-group than the S-group. The need for transfusion during adhesion dissection and the chest tube drainage were significantly lower in the T-group. These resulted from dissection of a smaller area in the T-group. However, the thoracotomy approach can only be applied in a limited number of cases, and conversion to median sternotomy will sometimes be required. To avoid this, we used the thoracotomy approach in selective cases. A right anterolateral thoracotomy approach was performed only in case of atrioventricular valve diseases or/and atrial septal defect.

In the T-group, we underwent one mitral valve replacement and one mitral valve repair without aortic cross-clamp. One patient had a previously grafted saphenous vein on the ascending aorta and the other had a previously grafted radial artery on the ascending aorta. As Umakanthan et al. reports the safety and reproducibility of clampless technique [6], we decided not to clamp the ascending aorta and open the left atrium as soon as possible after fibrillation. We also inserted a vent into the left ventricle and injected carbon dioxide continuously during the surgery. We successfully removed the air through root vent and weaned cardiopulmonary bypass. After the surgery, patient weaned ventilator smoothly and did not show any neurologic deficiency.

For cardiac re-operation, the reported mortality rate is 8–12.5% and post-operative bleeding rate is 2–4% [5,7-10]. We found a similar mortality rate. The most common cause of death was pneumonia in both groups. Bleeding was the second common cause of death in the S-group, but not in the T-group. There were three cases of post-operative bleeding, but these did not result in death in the T-group. In this study, 32 patients underwent cardiac re-operation for recurred tricuspid regurgitation. Given the high mortality rate of re-operation for recurred tricuspid regurgitation (up to 37%) [11,12], we believe that the mortality rate (7.2%) was not high compared to the previous studies.

### Limitations

This study is subject to the limitations inherent in retrospective work with observational data. There were no matched patients between the S-group and T-group, which caused difficulty in performing a simple comparison study. There was a limited number of operable valves and congenital heart diseases approachable through thoracotomy.

## Conclusions

Mortality and morbidity rates were favorable in both groups. Pneumonia was the most common cause of death in both groups. Re-operative approaches, thoracotomy and median sternotomy, for various cardiac diseases were safe and effective in terms of mortality, wound infection, bleeding, operation time, adhesion dissecting time, and bleeding control time. We were able to obtain a good visual field and perform safe surgery by applying the thoracotomy method in selective patients for cardiovascular re-operation.

## Competing interests

The authors declare that they have no competing interests.

## Authors’ contributions

JWC: drafting the manuscript, acquisition of data, analysis of data. HKC: study design, revising the manuscript. JSK: analysis of data, revising the manuscript.
